# Comparison of Titanium Dioxide and Zinc Oxide Photocatalysts for the Inactivation of *Escherichia coli* in Water Using Slurry and Rotating-Disk Photocatalytic Reactors

**DOI:** 10.1021/acs.iecr.3c00508

**Published:** 2023-08-04

**Authors:** Sean O’Neill, Jeanette M. C. Robertson, Valérie Héquet, Florent Chazarenc, Xinzhu Pang, Kathryn Ralphs, Nathan Skillen, Peter K. J. Robertson

**Affiliations:** † School of Chemistry and Chemical Engineering, Queen’s University Belfast, David Keir Building, Stranmillis Road, Belfast BT9 5GS, Ireland; ‡ IMT Atlantique, CNRS, GEPEA, UMR 6144, 4 rue Alfred Kastler, CS 20722, Nantes Cedex 3 44403, France; § School of Biological Sciences, Queen’s University Belfast, Chlorine Gardens, Belfast BT9 5DL, Ireland; ∥ Research Unit REVERSAAL, Centre INRAE Lyon-Grenoble, Auvergne-Rhône-Alpes, 5 Rue de la Doua, CS 20244, Villeurbanne Cedex 69625, France

## Abstract

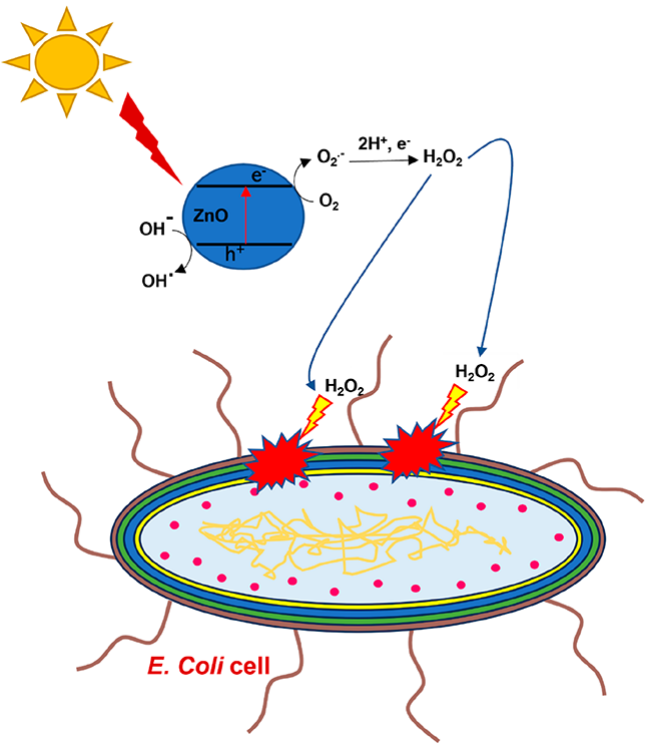

The application of photocatalysis for the disinfection of water has been extensively reported over the past 30 years. Titanium dioxide (TiO_2_) has been the most widely and successfully used photocatalyst to date; however, it is not without its limitations. Frequently observed long lag times, sometimes up to 60 min, before bacterial inactivation begins and the presence of residual microorganisms, for example, up to 10^4^ colony forming units, remaining after treatment are ongoing challenges with this particular photocatalyst. It is therefore important to find alternative photocatalysts that can address these issues. In this study, we compared the disinfection capacity of TiO_2_ with that of zinc oxide (ZnO) using *Escherichia coli* as a model organism in both a suspended and immobilized catalyst system. Our results showed that ZnO was superior to TiO_2_ in a number of areas. Not only were bacterial rates of destruction much quicker with ZnO, but no lag time was observed prior to inactivation in suspended systems. Furthermore, complete bacterial destruction was observed within the treatment times under investigation. The greater efficiency of ZnO is believed to be due to the decomposition of the bacterial cell wall being driven by hydrogen peroxide as opposed to hydroxyl radicals. The results reported in this paper show that ZnO is a more efficient and cost-effective photocatalyst than TiO_2_ and that it represents a viable alternative photocatalyst for water disinfection processes.

## Introduction

A serious worldwide issue today is the provision of effective water treatment technologies for the removal of pathogenic microorganisms from potable water. Waterborne diarrheal diseases such as cholera and dysentery are a leading cause of death among individuals living in low- and middle-income countries.^1^ Many of these deaths result from the lack of appropriate water and sanitation services. Access to safe and clean drinking water and sanitation has been recognized as a basic human right but unfortunately is still not available to all. Ongoing research into the development of innovative water treatment systems that can be deployed in areas of need is essential if we are to address this issue. Among promising water treatment technologies, heterogeneous photocatalysis, an advanced oxidation process, has been demonstrated to be a potential method for degrading a broad range of contaminants in water.^2^ Semiconductor materials have been reported extensively in the literature for their photocatalyst capabilities, which are due to their ability to perform concerted reduction and oxidation reactions.^3,4^ Following irradiation with light of an appropriate wavelength, an electron from the valence band of a semiconductor material will be promoted to the unoccupied conduction band, leaving behind a positive hole in the valence band. Subsequently, the excited electron may be used for reduction processes, while the positive hole can promote oxidation reactions, both of which results in the formation of reactive oxygen species (ROS), such as superoxide (O_2_^•–^) and hydroxyl radicals (^•^OH). O_2_^•–^ generated via the conduction band reaction following protonation and further reduction can generate hydrogen peroxide (H_2_O_2_). These ROS can then break down a broad range of chemical and biological contaminants in water.^2,5^^•^OH have a redox potential of 2.8 V versus normal hydrogen electrode (NHE) but have a short half-life of around 10^–9^ s.^5^ H_2_O_2_ has a lower redox potential of 1.8 V vs NHE but has a longer half-life, which could provide H_2_O_2_ with a greater potential to interact with, and hence degrade, the contaminants.^5^

While there are a broad range of semiconductor materials that have displayed photocatalytic activity, titanium dioxide (TiO_2_) has been one of the most extensively researched due to its stable structure, nontoxicity, and high photocatalytic activity.^6,7^ Since the initial pioneering work by Ireland et al. in 1993,^8^ the use of TiO_2_ for the treatment of water contaminated by a broad range of microorganisms has been reported.^9−13^ While this photocatalyst has been clearly demonstrated to be efficacious in microbial inactivation, previous studies have reported the observation of a lag period before any significant inactivation takes place.^14−16^ Furthermore, complete removal of all microorganisms is rarely reported, with residual numbers of surviving microorganisms often being observed.^17,18^ While generally small in number, residual microorganisms that have survived the disinfection treatment process may continue to grow and increase in density. Moreover, complete removal of all microorganisms is required in order to meet the World Health Organisation (WHO) guidelines for the microbial quality of drinking water.^19^

Consequently, these limitations have inhibited the practical application of TiO_2_ photocatalysts for water treatment processes. Zinc oxide (ZnO) is another semiconductor photocatalyst material that exhibits photocatalytic characteristics similar to those of TiO_2_,^20^ but it has been shown to have a higher quantum efficiency than TiO_2_ for several photocatalytic processes.^21^ ZnO nanoparticles are gaining increasing popularity for use in many industrial and biomedical applications. This is due to a number of different properties including low cost, low toxicity, and biocompatibility.^22^ Their use, however, in water disinfection studies has been limited due to toxicity concerns in aquatic environments.^23,24^ The risks of ZnO nanoparticles include the potential threat to nontarget organisms like fish and crustaceans.^25^ In their comprehensive review on the potential ecotoxicity of ZnO nanoparticles, Ma et al. considered the impact of these particles on a broad range of potential targets in the environment including bacteria and algae as well as both terrestrial and aquatic vertebrate and invertebrate species.^26^ They reported that the toxicity presented by this material greatly depended on the species under investigation, but there were still gaps in the overall detailed understanding of the ecotoxicity of ZnO nanoparticles in the environment.^26^ Consequently, this highlights the importance of the effective separation of ZnO particles from treated water in slurry photocatalytic reactor systems prior to use or discharge. Alternatively, the use of reactors where the ZnO photocatalyst is immobilized, such as that detailed in this paper, would minimize the risks of emission of particulate ZnO material into the environment. While previous work has compared both photocatalysts for the water treatment of chemical pollutants,^27^ this research presents a comparative investigation of both slurry and immobilized TiO_2_ and ZnO photocatalytic systems for their efficacy for the inactivation of bacteria. Bacterial contaminants are orders of magnitude larger and more complex than organic pollutants. Examining how TiO_2_ and ZnO interact with bacteria would provide insight into the different processes by which these two photocatalysts act to inactivate microorganisms. In this investigation, the performances of both suspended and immobilized photocatalyst systems utilizing ZnO and TiO_2_ materials for the inactivation of *Escherichia coli* have been compared.

## Experimental Section

### Preparation of Bacterial Cultures

*E. coli* K12 was stored on Protect beads (Technical Service Consultants) at −20 °C. Cultures were prepared by removing a bead from the frozen Protect vial and inoculating it into 10 mL of nutrient broth. The broth was then incubated at 37 °C for 24 h. The culture was washed by centrifugation at 3500 rpm followed by replacement of the nutrient broth with 10 mL of sterile distilled water. This process was repeated two more times. The optical density of the washed culture was adjusted to 0.5 at λ = 600 nm, which is approximately 10^8^ CFU mL^–1^ (CFU = colony forming units), using a Helios Omega UV–Visible spectrometer (Thermo Scientific).

### Photocatalytic Disinfection Studies Using Suspended Photocatalyst Powders

**Figure 1:**
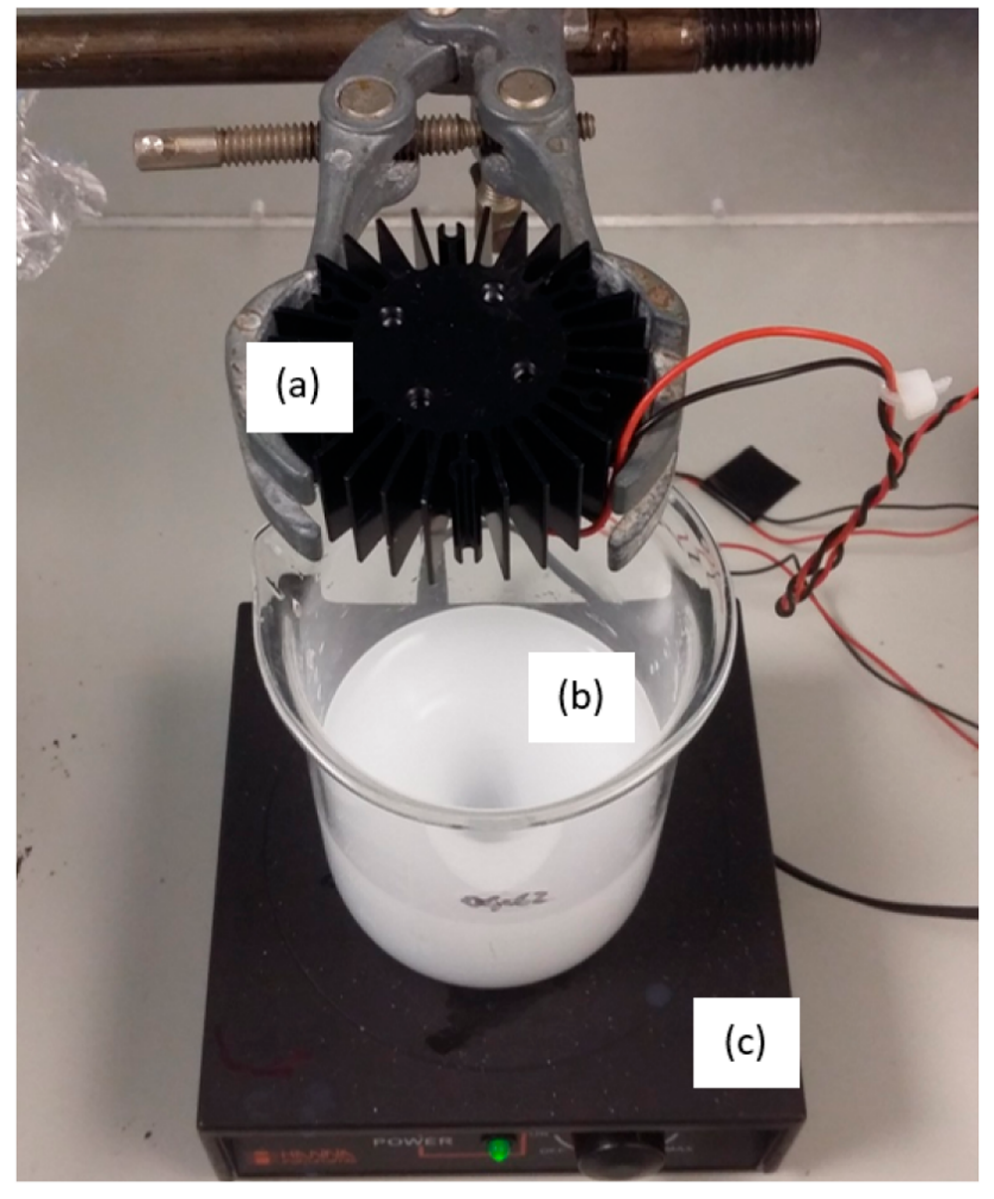
Photocatalysis setup for inactivating *E. coli* in photocatalyst suspended systems using a UV LED: (a) UV LED; (b) 100 mL of suspended catalyst; (c) stirrer plate.

TiO_2_ (Evonik P25) and ZnO (Fisher) photocatalyst materials were used as supplied. The characterization data for the ZnO and TiO_2_ photocatalytic materials are provided in the Supporting Information. Photocatalysis experiments were conducted in 250 mL sterile beakers at room temperature (Figure 1). A 1 mL aliquot of the washed bacterial culture was added to 100 mL of a 1 g L^–1^ suspension of TiO_2_ in sterile distilled water. Irradiation of the unit was provided by a UV light-emitting diode (LED; LZ1-10UV00-0000, LED Engin Europe) mounted onto a 50 × 20 mm heatsink (ILA[1]HSINK-STAR, Intelligent LED solutions), which had a spectral output of 350–400 nm with a peak wavelength of 370 nm and a viewing angle of 70° (the spectral output of the LED is presented in the Supporting Information, specifically section S4). A variable direct-current power supply supplied the voltage to give a forward voltage (VF) of 3.5 dcV and a forward current (IF) of 0.3 A, which gave an overall power of 1.05 W (the spectral output of the LED strips is presented in the Supporting Information). The LED unit was positioned at a height of 10 cm above the reaction beaker. Control solutions consisting of 1 mL of bacterial culture in 100 mL of sterile distilled water only (UV control) and 1 mL of bacterial culture in 100 mL of a 1 g L^–1^ suspension of TiO_2_ in sterile distilled water, which was not irradiated and was kept in the dark (dark control), were also prepared. All reaction beakers were continuously stirred for the duration of the experimental period to improve mass transfer and to help prevent settling of the catalyst at the bottom of the reaction beakers.

The same experimental procedure was conducted for experiments examining the photocatalytic disinfection properties of ZnO, but this time a 1 g L^–1^ suspension of ZnO was used, instead of TiO_2_. This is a typical loading for photocatalytic reactions in laboratory-based suspended systems. Samples (1 mL aliquots) were collected from the reaction beakers at 30 min intervals. These were diluted 10-fold in sterile distilled water, and viable bacterial counts were performed on nutrient agar using the method of Miles and Misra.^28^

### Preparation of Immobilized Photocatalyst

Photocatalyst films were prepared and coated onto one side only of 12 cm borosilicate glass disks using the sol–gel method of Mills et al.^29^ The side to be coated with the catalyst film was initially sandblasted to improve the morphology of the disk surface and consequently enhance the adhesion of the photocatalyst to the glass surface. For preparation of the initial catalyst films, a mixture was produced using 1 g of either TiO_2_ (Evonik P25) or ZnO (Fisher) powder, which was then added to 0.01 g of KD-1 dispersant (Croda), 10 mL of isopropyl alcohol (Sigma-Aldrich), and 5 g of poly(ethylene glycol) (PEG; Sigma-Aldrich). For both film types, the components were added together in a beaker and ultrasonicated for 15 min; this was followed by mixing with a magnetic stirrer for 30 min to produce a uniform suspension. The suspension was then carefully applied to the sandblasted side of the glass disks using a small paintbrush, and the glass disks were placed in an oven at 50 °C for 20 min to dry. This process was repeated four more times, resulting in the application of 0.5 g of photocatalyst to the surface of each glass disk. This was determined by the weight difference of the glass disks before and after catalyst application. When the film had fully dried, the disks were calcined in a furnace at 500 °C for 1 h and allowed to cool before use in photocatalysis experiments.

### Photocatalytic Disinfection Studies Using Immobilized Photocatalysts

**Figure 2:**
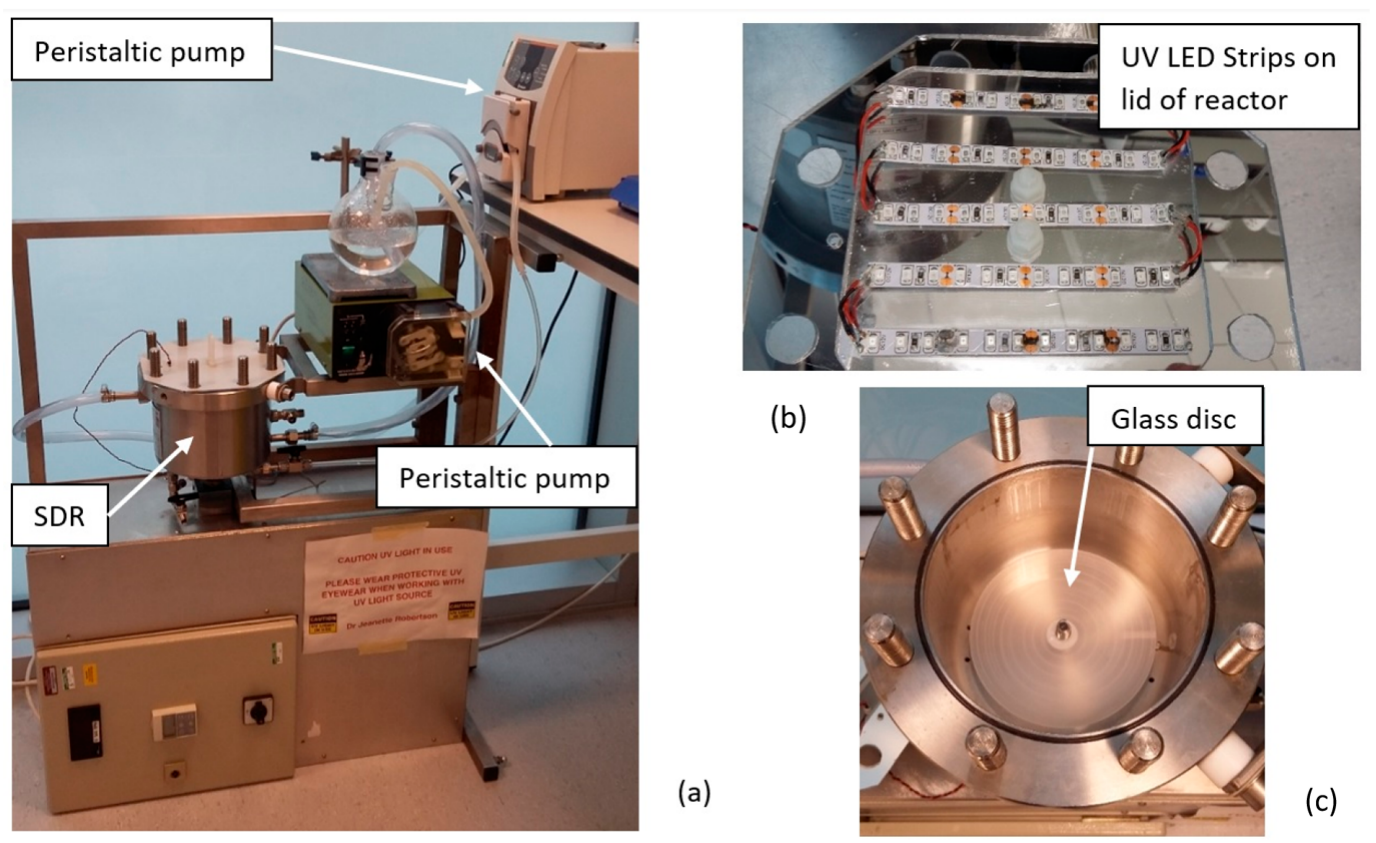
(a) SDR. A peristaltic pump circulates the water around the vessel and cooling water around the water jacket. (b) UV LED strips attached to the bottom of the lid of the reactor. (c) Image of the internal view of the SDR with a TiO_2_-coated disk held in place by the central metal rod.

Photocatalytic disinfection studies with immobilized photocatalysts were performed in a spinning disk reactor (SDR; Figure 2a). In this reactor, the immobilized photocatalyst (either TiO_2_ or ZnO coated to borosilicate glass) was secured to a rotating rod in the center of the reactor (Figure 2c).

Irradiation was provided by a five UV LED strip array constructed from UV LEDs (Lighting Will), which provided irradiation within the SDR. The LEDs had a peak wavelength in the range of 365–370 nm and were operated at VF = 12.0 dcV and IF = 1.1 A, which gave an overall electrical power of 13.2 W (Figure 2b; the spectral output of the LED strips is presented in the Supporting Information, specifically section S4). Each strip contained 12 LEDs, giving a total of 60 LEDS that were mounted to the lid of the reactor unit. For disinfection experiments, a 1 mL aliquot of washed bacterial culture was added to 1000 mL of sterile distilled water in the main reactor vessel, the lid of the vessel was secured, and the UV LEDs were switched on. The water was circulated through the reactor via a peristaltic pump at 8 mL s^–1^ to improve the mass transfer during experiments. The rotation of the catalyst-coated disks in the SDR was maintained at 140 rpm because previous research had shown this to be the optimal speed. A water jacket surrounded the SDR, and the water in this jacket was circulated by a second peristaltic pump. Control experiments were also undertaken; for UV only, control experiments of an uncoated glass disk were employed in the SDR, and dark controls were performed using the catalyst-coated disk but in the absence of any light. Samples (3 mL aliquots) were taken from the SDR at specified time intervals and processed for viable counts, as outlined in Photocatalytic Disinfection Studies Using Suspended Photocatalyst Powders.

### Assessment of H_2_O_2_ Generation by TiO_2_ and ZnO Photocatalysts

The ZnO or TiO_2_ photocatalyst (50 mg) was suspended in a 250 mL glass beaker containing 100 mL of a 0.1 M methanol solution (Sigma-Aldrich) and gently mixed using a magnetic stirrer. A UV LED light (Series ILH-Xx01-Sxxx-SC211-WIR200, Intelligent LED Solutions) with a peak wavelength at 370 nm and a 65° viewing angle was placed directly above the beaker to provide UV light (*I* = 0.25 A and *V* = 14 V). The H_2_O_2_ concentrations in the samples were determined using the horseradish peroxidase (HRP; Alfa Aesar)-catalyzed stoichiometric dimerization of a *p*-hydroxyphenylacetic acid (POHPAA; Tokyo Chemical Industry) method, which yields a fluorescent product (λ_ex_ = 315 nm; λ_em_ = 406 nm).^30^ A total of 8 mg of POHPAA and 2 mg of HRP were dissolved in Tris buffer (25 mL, 1.0 M, pH 8.8), followed by the addition of a 2 mL sample of the test solution (diluted when required). A total of 0.25 mL of the fluorescent solutions were subsequently analyzed using fluorescence spectroscopy (PerkinElmer LS 50 B luminescence spectrometry fluorimeter; λ_ex_ = 315 nm; λ_em_ = 406 nm) following a 30 min reaction time. The H_2_O_2_ concentrations were calculated from a calibration curve prepared from known H_2_O_2_ concentrations.

## Results and Discussion

### Photocatalytic Disinfection Studies Using Photocatalysts in a Suspended System

**Figure 3:**
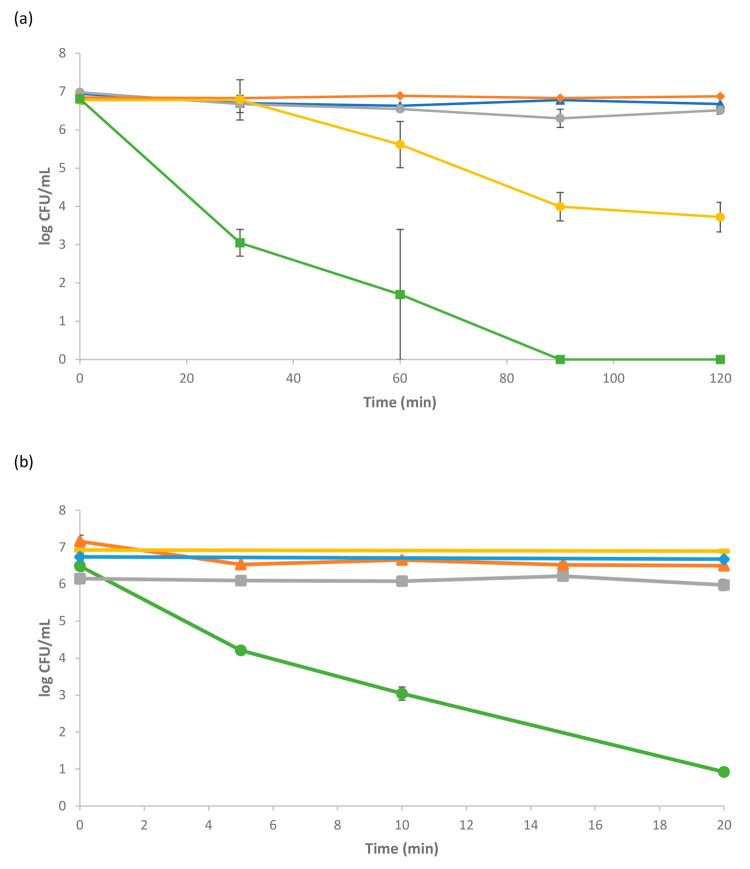
Photocatalytic inactivation of *E. coli* using a suspended system of ZnO or TiO_2_ for (a) a 120 min illumination period and (b) a 20 min illumination period: (orange ▲) light control; (gray ■) ZnO dark control; (blue ◆) TiO_2_ dark control; (green ●) photocatalysis with ZnO; (yellow —) photocatalysis with TiO_2_. Three replicate experiments were performed.

Figure 3 shows the findings from the disinfection experiments using both TiO_2_ and ZnO photocatalyst materials. As shown in Figure 3a, inactivation of *E. coli* did not occur during the first 30 min of irradiation using the TiO_2_ photocatalyst. After this period, however, there was a rapid reduction, of up to 3 log orders, in bacterial numbers for up to 90 min of photocatalysis time. Thereafter, the bacterial numbers continued to fall but at a much slower rate. At the end of the experimental period (120 min), around 10^4^ CFU mL^–1^ of bacteria remained. When the bacterial culture was irradiated in the presence of the ZnO photocatalyst, a rapid and immediate reduction in viable bacterial numbers was observed from the start of irradiation, and no surviving bacteria were detected after 90 min photocatalysis. No significant inactivation of *E. coli* was observed in the dark or light control experiments for either photocatalyst material.

Overall, the results in Figure 3a show that ZnO is a much more efficient photocatalyst for inactivating bacteria than TiO_2_. Complete inactivation of all bacteria within 90 min of treatment time was observed when ZnO was used, whereas around 10^4^ CFU mL^–1^ of bacteria remained in the reaction vessel at the end of the 120 min of treatment time when TiO_2_ was employed. Furthermore, the rate at which ZnO deactivated bacteria was also significantly faster than that of TiO_2_. Bacterial inactivation with the ZnO photocatalyst began almost immediately, whereas when TiO_2_ was used, there was a lag period of around 30 min before any bacterial inactivation was observed. Within 90 min of photocatalysis treatment time, there was complete inactivation of *E. coli* using the ZnO material, while at the same time period, only 10^3^ CFU mL^–1^ of the bacteria had been destroyed when the TiO_2_ photocatalyst was used, with 10^4^ CFU mL^–1^ remaining. This lag in bacterial inactivation has been reported in other studies using TiO_2_, and it has been proposed that it is a result of the main type of ROS produced when TiO_2_ materials are irradiated.^14−16^ Typically, TiO_2_ photocatalysts produce large quantities of ^•^OH,^31,32^ whereas H_2_O_2_ is produced in lower quantities on this material (<0.2 mM).^32^ This is thought to be due to H_2_O_2_ generated via the conduction band reaction subsequently undergoing photocatalytic degradation via conduction band electrons to generate further ^•^OH.^32^ Sontakke and co-workers previously reported enhancement of the photocatalytic deactivation of *E. coli* using a silver-modified TiO_2_ photocatalyst.^33^ It is believed that this enhancement is due to the added peroxide being broken down to ^•^OH and a hydroxide ion through reaction with photogenerated conduction band electrons. While both species are strongly oxidizing, ^•^OH are highly unstable and their lifetime in water has been reported to be around 10^–6^ s.^31^ Consequently, if these radicals do not interact quickly with the target bacteria, they are likely to undergo dimerization or decompose before they can exert any damaging effects on the bacteria.^32^ If ^•^OH dimerize to form H_2_O_2_ on the surface of TiO_2_, the peroxide can subsequently react with a photogenerated electron in the conduction band to produce ^•^OH and a hydroxide ion. This reduction reaction between the conduction band electrons and H_2_O_2_, however, does not occur on ZnO materials.^21^^•^OH can also rapidly react with other species in water, such as trace organic materials or inorganic ions, such as chloride. It has been previously reported that the mode of action of bacterial inactivation by photocatalysis is a result of the cell wall being ruptured by ROS generated by the photocatalyst.^32^ If there are not sufficient quantities of the ROS available to attack the cell wall, this initial process will be slow, and there will be a potential lag before the initial inactivation of the bacteria is observed following initiation of the photocatalytic reaction. Because ^•^OH are relatively short-lived, they rapidly decompose as detailed above. Consequently, this may mean that significant quantities of these species may not be generated to result in a sustained attack on the bacteria cell wall, and hence this may explain the lag period observed when using TiO_2_ photocatalysts in this study. Conversely, ZnO produces significant quantities of H_2_O_2_ during photocatalytic reactions.^34^ H_2_O_2_ is a much more stable species in water and is more likely to accumulate once the photocatalysis is started. Because there is potentially a greater concentration of peroxide to damage the bacteria cell wall, this may be the reason why a more immediate and rapid inactivation of *E. coli* was observed using the ZnO photocatalyst in this study. Raffellini et al. reported a detailed study of the effectiveness of H_2_O_2_ for the inactivation of *E. coli* and key parameters that influenced the kinetics of the inactivation process.^35^ Due to the rapid inactivation observed, a second experiment was undertaken in an attempt to examine the reaction kinetics taking place in the initial stages of photocatalytic disinfection with ZnO. Figure 3b shows that for ZnO bacterial inactivation was detected within the first 5 min of photocatalyst irradiation and that this was almost complete after 20 min. The existence of the lag in the initiation of bacterial inactivation has been a significant challenge for the practical application of the photocatalytic disinfection process with TiO_2_, particularly when compared to conventional water disinfection techniques such as chlorination.^14−16^ This work has, however, shown that this problem may be overcome using a ZnO photocatalyst, which induced an immediate and rapid inactivation of *E. coli*. No bacterial inactivation occurred with either the TiO_2_ or ZnO light and dark controls.

**Figure 4:**
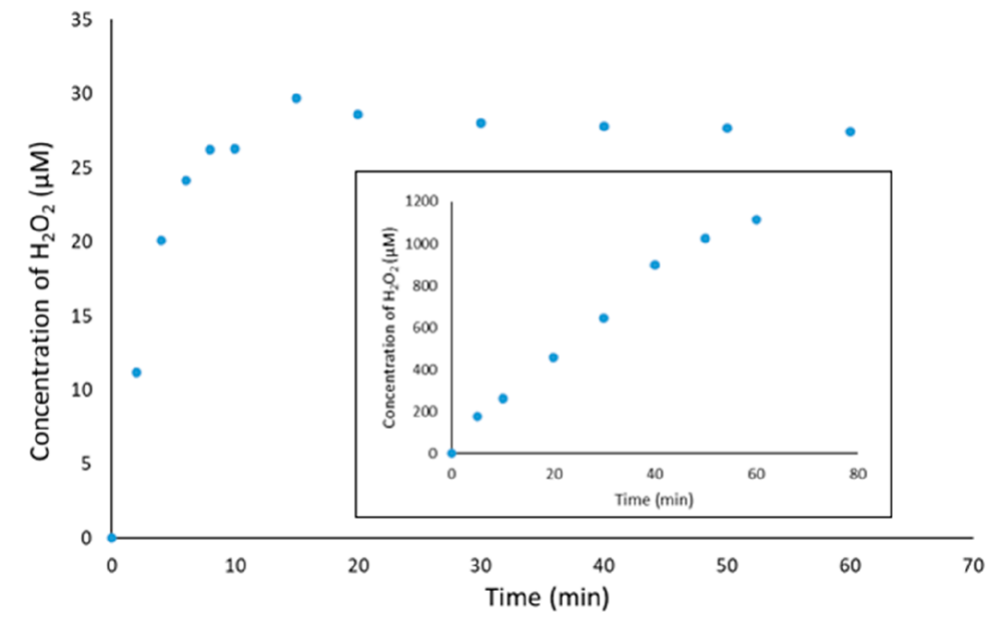
Generation of H_2_O_2_ using a TiO_2_ photocatalyst from a methanol/O_2_ system (inset: generation of H_2_O_2_ using a ZnO photocatalyst).

Das et al.^36^ investigated the use of scavengers to determine the relative importance of ^•^OH and H_2_O_2_ on the photocatalytic activity of ZnO for *E. coli* inactivation. Their study showed that, with the removal of ^•^OH, the inactivation efficiency of ZnO fell by ≈55%, which indicated that ^•^OH had a significant role in the photocatalytic activity. When a H_2_O_2_ scavenger [iron(II) ethylenediaminetetraacetic acid (Fe^II^-EDTA)] was added to the system, a ≈86% decline in the inactivation efficiency was, however, observed.^33^ This research indicates that H_2_O_2_ is a key ROS involved in the inactivation of *E. coli* with ZnO. The production of H_2_O_2_ has been shown to correlate with the available surface area of ZnO, which results in more oxygen species on the surface and a higher antibacterial activity of the catalyst.^37^ A particular advantage of H_2_O_2_ over other ROS is the capability of H_2_O_2_ to enter the cell membrane of bacteria and damage the internal structures, while ROS like ^•^OH and O_2_^•–^ cannot infiltrate the cell membrane.^34^ The comparative efficiencies of TiO_2_ and ZnO in generating H_2_O_2_ were estimated, as shown in Figure 4, and the results showed that the yield of H_2_O_2_ on illuminated ZnO was more than 300 times greater than that for TiO_2_, showing that ZnO has a greater ability to reduce oxygen into H_2_O_2_ via the conduction band reaction (Figure 4).

While the role of ROS in bacterial inactivation is shown to have an important one for all photocatalysts, the superior bacterial inactivation properties observed by ZnO here, however, may not be limited to the actions of ROS alone but may be due to a combination of several antibacterial mechanisms that have been reported to occur with this photocatalyst material.^22,38^ ZnO is capable of releasing Zn^2+^ at high pH levels, following oxidation. Zn^2+^ ions have been demonstrated to be toxic to a range of microbial species. It is important to note that neither of these processes was observed in our study for the inactivation of *E. coli* because no bacterial inactivation was observed in the dark control experiments. ZnO has, however, been reported in the literature to degrade under irradiation, and Zn^2+^ could be released during photocatalysis. Consequently, a control experiment was performed, irradiating a ZnO suspension under the same conditions that the bacterial inactivation work was performed. Using inductively coupled plasma (ICP) analysis of the solution following photocatalysis, it was shown that around 3 ppm Zn was detected in the water following 120 min of photocatalysis. While the WHO has not set a “health-based guideline” limit for Zn in drinking water, it has highlighted the threshold level for Zn at which an unpleasant taste is observed as 4 ppm.^39^ Consequently, a level of 3 ppm is suggested for drinking water. While no specific method of bacterial inactivation by photocatalysts has yet been identified, observed alterations in the cell structure during photocatalytic treatment have led to the generally accepted theory that the ROS produced during photocatalytic reactions cause direct cell membrane damage followed by leakage of intracellular contents.^40−43^ The faster these ROS are produced and the more stable they are, the greater chances there are of significant cell damage occurring and, consequently, cell death.

**Figure 5:**
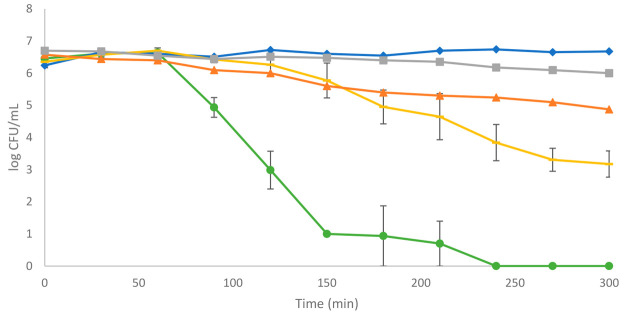
Photocatalytic inactivation of *E. coli* in the SDR using borosilicate disks coated with either a ZnO or TiO_2_ film with the addition of PEG to aid bonding: (blue ◆) dark control; (gray ■) ZnO dark control; (orange ▲) light control; (yellow —) photocatalysis with a TiO_2_ disk; (green ●) photocatalysis with a ZnO disk. Three replicate experiments were performed.

While slurry reactors have been shown to be much more efficient for bacterial disinfection studies, the post-treatment removal and recovery of catalyst required for this type of system is another limiting factor for larger-scale applications of this technology. Consequently, in many up-scaled systems, the photocatalyst is immobilized onto a suitable substrate to avoid any post-treatment catalyst recovery steps.^44−46^ The downside with immobilized systems, however, is that treatment times tend to be longer due to issues around mass-transport limitations in the reactors and also a relatively lower quantity of active photocatalyst surface areas. It is important for any photocatalytic reactor that the catalyst and target species are in sufficient contact with one another for an appropriate time period to allow bacterial inactivation to take place. One approach where these limitations may be minimized is in the use of a rotating SDR.^47^ Consequently, in this work, the efficacy of both the TiO_2_ and ZnO photocatalysts was examined in an SDR. Each of the photocatalysts were immobilized on glass disks that were deployed in the SDR and assessed for the inactivation capacity of *E. coli* (Figure 5).

From Figure 5, it can be seen that inactivation of *E. coli* also occurred using the immobilized catalyst systems, and as with the previously suspended catalyst systems, this was more efficient with the ZnO photocatalyst. Complete inactivation of *E. coli* was achieved within 250 min of irradiation time with the ZnO-coated disk, whereas with the TiO_2_-coated disk, around 10^3^ CFU mL^–1^ of *E. coli* remained after 300 min of irradiation time. No bacterial inactivation was observed within the dark controls, and a minor inactivation, approximately 1 order of magnitude, was observed with the light controls. Unsurprisingly, the immobilized systems in the SDR had slower kinetics compared to the suspended systems due to the relatively smaller active photocatalyst surface area compared to the slurry reactor systems.^43^ It can also be seen in the figure that an initial lag time was observed prior to the photocatalytic inactivation of the bacteria for both photocatalyst materials. This is probably due to the relatively smaller quantity of active photocatalyst that is available for the inactivation process compared to that available in the suspended catalyst system, which hence generated lower quantities of the ROS, i.e., ^•^OH for TiO_2_ and H_2_O_2_ for the ZnO material. It can be seen, however, that while this lag time existed for both photocatalyst materials, once the disinfection process started, the overall kinetics for the ZnO system were significantly faster than that for the TiO_2_ photocatalyst.

## Conclusions

ZnO has been demonstrated to be a significantly more effective photocatalyst than TiO_2_ for the inactivation of *E. coli* in both suspended and immobilized photocatalyst systems. In the suspended catalyst systems, bacterial inactivation with ZnO was immediate and rapid, whereas with TiO_2_, a lag period, before any bacteria inactivation began, was evident. This lag is thought to be due to the difference in the main ROS produced by both photocatalysts and their ability to induce cell wall damage in the target bacteria. TiO_2_ produces highly oxidative but highly unstable ^•^OH, which requires more time to bring about sufficient bacterial cell wall damage to induce cell death. On the other hand, the main ROS produced by ZnO is thought to be H_2_O_2_, a more stable ROS with better microbial cell-wall-penetrating properties. While we have demonstrated that ZnO photocatalytically generates more H_2_O_2_ than TiO_2_, further research, however, is needed to ascertain the exact mechanisms by which ZnO generates ROS and how these species interact with contaminants. Overall, the lack of any lag period in the initiation of bacterial inactivation and the overall faster reaction kinetics observed with ZnO demonstrate the superior activity of this photocatalyst in bacteria disinfection studies, compared to TiO_2_ for suspended catalyst systems. In the immobilized catalyst reactor, a slight lag in bacterial inactivation was observed with the ZnO photocatalyst; this was, however, significantly shorter than that observed with the TiO_2_ material. Furthermore, as with the suspended catalyst system, complete bacterial inactivation was achieved using ZnO materials, while with TiO_2_, 10^3^ CFU remained in the same reaction period.

## Supporting Information Available

Data and findings presented in this paper supported by additional work including Brunauer–Emmett–Teller (BET) surface area, X-ray diffraction, and UV–vis spectral analysis of the TiO_2_ and ZnO photocatalysts, spectral outputs of the LED sources, and ICP analysis of the treated water samples (PDF)
