# Supplementary material for: Comparison of Titanium Dioxide and Zinc Oxide Photocatalysts for the Inactivation of Escherichia coli in Water Using Slurry and Rotating-Disk Photocatalytic Reactors
Source: Ind Eng Chem Res. 2023 Aug 4;62(45):18952–9. doi: 10.1021/acs.iecr.3c00508 (PMC10655038; doi:10.1021/acs.iecr.3c00508)
Supplement: Supplementary file 1 — ie3c00508_si_001.pdf [file ie3c00508_si_001.pdf]

Supporting Information

A Comparison of Titanium Dioxide and Zinc Oxide Photocatalysts for the Inactivation of *Escherichia coli* in Water using Slurry and Rotating Disc Photocatalytic Reactors.

Sean O'Neill [a,b], Jeanette M.C. Robertson [c], Valérie Héquet [b], Florent Chazarenc [d], Xinzhu Pang[a], Kathryn Ralphs[a], Nathan Skillen[a], and Peter K.J. Robertson [a*].

[a]School of Chemistry and Chemical Engineering, Queen's University Belfast, David Keir Building, Stranmillis Road, Belfast, BT9 5GS.
[b]IMT Atlantique, CNRS, GEPEA, UMR 6144, 4 rue Alfred Kastler, CS 20722, 44403 Nantes Cedex 3, France.
[c]School of Biological Sciences, Queen's University Belfast, Chlorine Gardens, Belfast, BT9 5DL
[d]Research Unit REVERSAAL, Centre INRAE Lyon-Grenoble, Auvergne-Rhône-Alpes, 5 Rue de la Doua, CS 20244/69625 Villeurbanne Cedex, France

* Corresponding author.
p.robertson@qub.ac.uk

S1, BET surface area of TiO_2 and ZnO

The BET surface area is offered as a service via By Analytical Services and Environmental Projects (ASEP) in QUB, using Micromeritics Tristar Brunauer–Emmett–Teller (BET) 3020.

TiO_2: 51.98 m^2/g

ZnO: 4.98 m^2/g

S2, XRD analysis of TiO_2 and ZnO

XRD data was recorded in Bruker AXS, 5465 East Cheryl Parkway, USA (40 kV, 25 mA) form 5° to 90°.

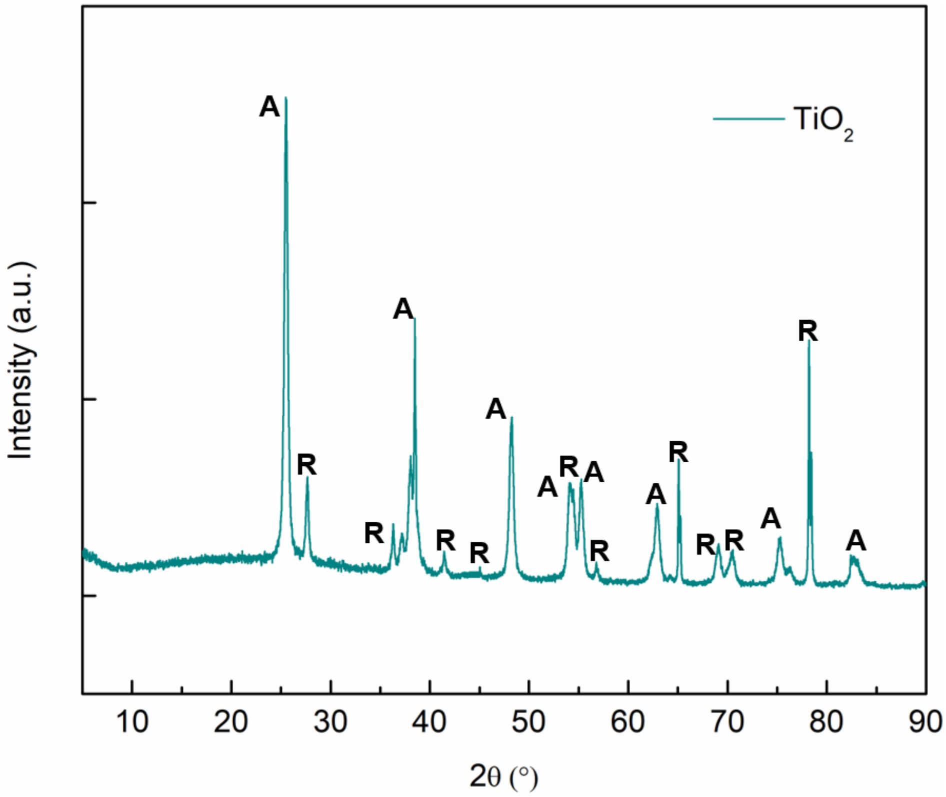

Figure S1. XRD Spectrum of TiO_2 photocatalyst.

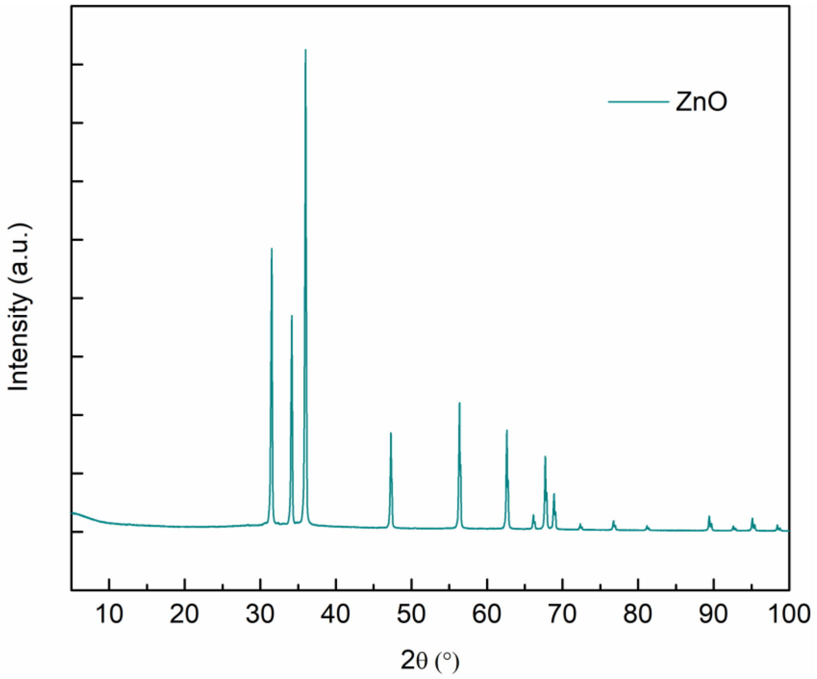

Figure S2. XRD Spectrum of ZnO photocatalyst.

S3, Optical analysis of TiO_2 and ZnO

Diffuse reflectance UV-vis spectra were measured on a UV-vis spectrophotometer (KONICA MINOLTA SENSING, INC. CM-2500d) with $BaSO_4$ as a reference. UV-vis spectra were used to calculate the band gap. The band gap of TiO_2 and ZnO was calculated as 3.28 and 3.25 eV, respectively.

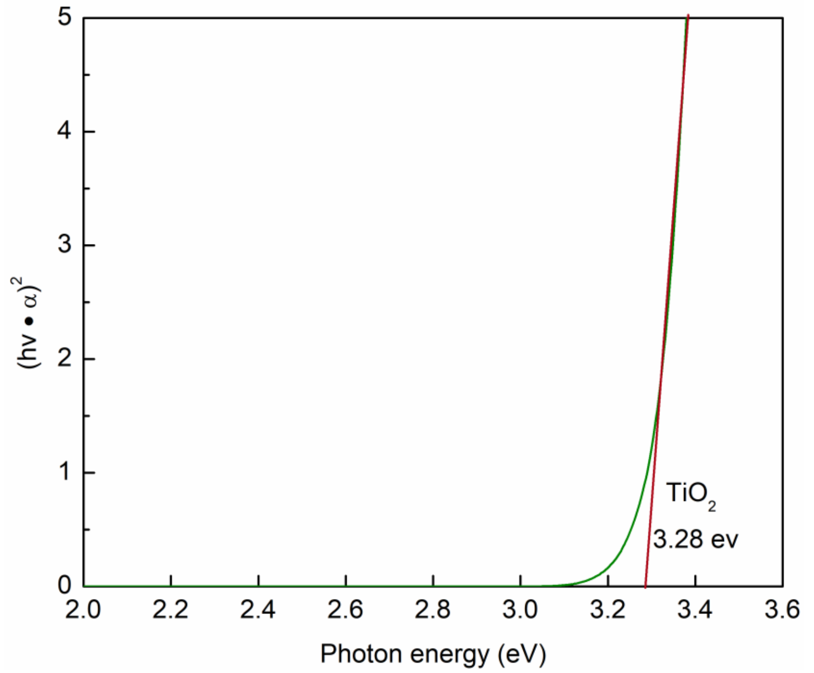

Figure S3. Diffuse Reflectance UV/Visible Spectrum of TiO_2 photocatalyst.

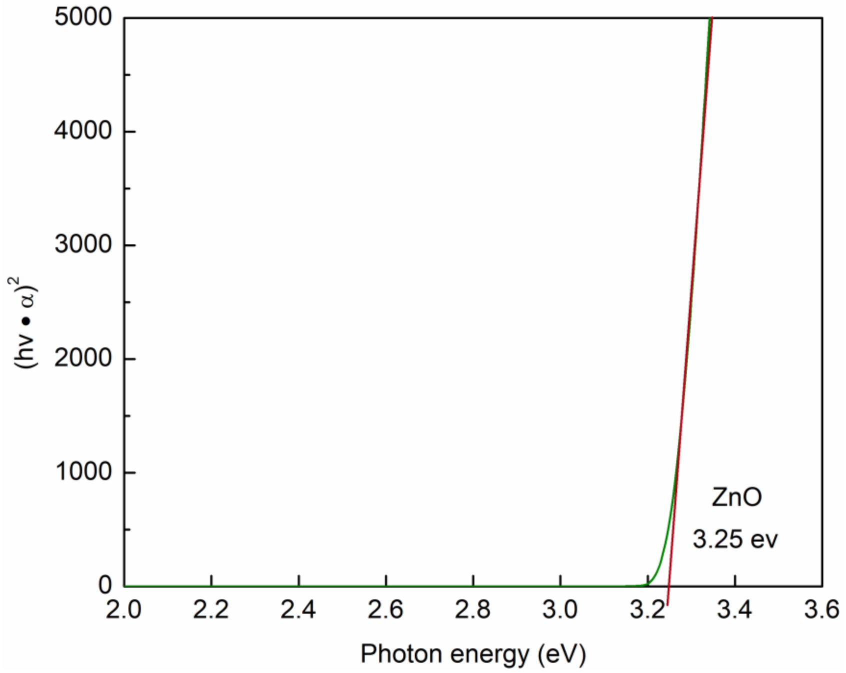

Figure S3. Diffuse Reflectance UV/Visible Spectrum of ZnO photocatalyst.

S4 Spectral Output of LED Sources.

S4.1. LZ1-10UV00- 0000, LED Engin Europe 370nm UV LED Spectral Range 350nm to 400nm. Data sheet including spectrum of LED can be accessed at https://octopart.com/datasheet/lz1-10uv00-0000-ledengin-30947079?utm_source=bing&utm_medium=cpc&utm_campaign=b_cpc_intl_search_dsa_english_en_usd_datasheets&utm_term=datasheet&utm_content=Intl%20Datasheet%20DSA

S4.2. Lighting Will 365-370 nm LED Strip Array.

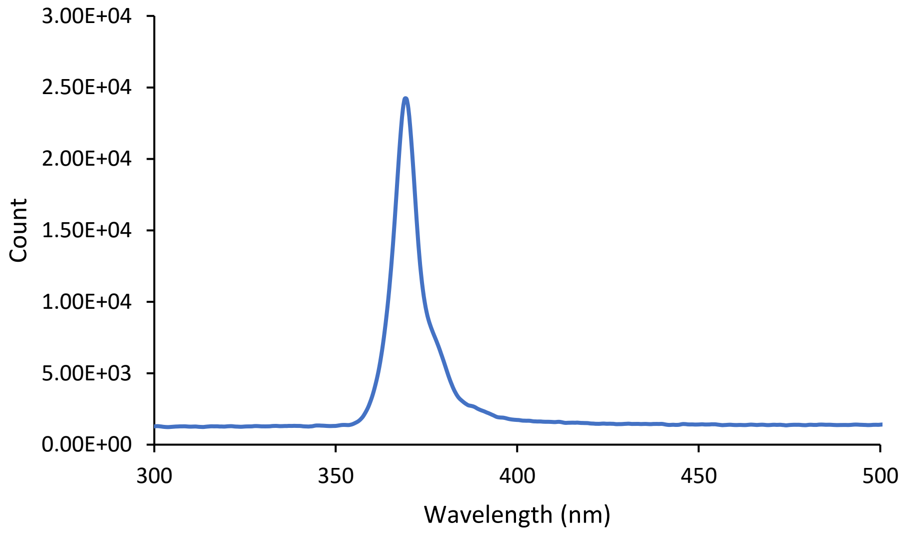

Figure S5. Spectral Output of Lighting Will 365-370 nm LED Strip Array.

S5. Determination of Zn Content in Water following Photocatalysis.

Following photocatalysis for 120 minutes the treated water was assessed using ICP Spectroscopy to determine the levels of Zn present potentially resulting from photocorrosion of the ZnO photocatalyst. The analysis was performed using an Agilent OES 5100 Inductively Coupled Plasmas Spectrometer, performed by Analytical Services and Environmental Projects (ASEP) in QUB. The samples were assessed in duplicate, and the levels of Zn detected were 3.1 and 3.2 ppm respectively.
